# Development of bio-based material from the *Moringa oleifera* and its bio-coagulation kinetic modeling–A sustainable approach to treat the wastewater

**DOI:** 10.1016/j.heliyon.2022.e10447

**Published:** 2022-09-01

**Authors:** Vaishali Varsani, Suhas J. Vyas, Dushyant R. Dudhagara

**Affiliations:** Department of Life Sciences, Bhakta Kavi Narsinh Mehta University, Khadiya-362263, Junagadh, Gujarat, India

**Keywords:** *M. oleifera* seeds, Bio-coagulants, Surface functionalized, Turbidity kinetics

## Abstract

The development of bio-coagulants from *Moringa oleifera* seeds for sewage wastewater treatment has been investigated. The prepared bio-coagulant was treated with distilled water, HCl, NaOH, and NaCl to surface-functionalize the *M. oleifera* seed powder (MOSP). The bio-coagulant performance was investigated by monitoring the reduction of turbidity, EC, pH, TS, BOD, and COD from the wastewater. SEM, EDAX, and FTIR characterized the native and functionalized MOSP bio-coagulants. The HCl treated MOSP was found to be effective and have good coagulation activity (∼90%) compared to natural and other MOSPs. The turbidity removal by all the MOPS conformed to the WHO acceptable limit of finished water. Hence, maximum turbidity reduction was recorded in HCl > NaCl > NaOH > Natural (distilled water) treated MOSP. The pseudo-first and second-order kinetics rate also showed the effectiveness against turbidity reduction in municipal sewage water. Hence, the prepared MOSP bio-coagulants could be suitable for primary water treatments.

## 1 Introduction

Wastewater disposal is the main issue being faced by all of us. In India presently, only about 10% of the wastewater generated is treated; most is released into surface water (Varsha et al., 2010). Sanitary sewage or municipal wastewater refers to domestic sewage discharged from commercial, institutional, and similar facilities. Municipal sewage water includes typical waste from sinks, faucets, toilets, showers, floor cleaning, laundry, and other garbage that people may pour down the drain system (Kamal et al., 2012). Therefore, sewage water contains many hazardous and toxic compounds. The rapid expansion of such water bodies worldwide has resulted in the creation of various types of waste, including various forms of dissolved and undissolved substances, and consequently increased water pollution (Dao et al., 2016). Suppose these ground surface water bodies are mixed with drinking water or freshwater, such as rivers, ponds, or streams. In that case, it causes harmful effects on living organisms, directly affecting the food chain and the whole ecosystem. Consequently, it is required to manage these kinds of wastewater systems.

Therefore, a wide range of technologies such as coagulation or flocculation, reduction or oxidation (Ai-Min et al., 2006), filtration (Fane et al., 2015), and anaerobic methods (Jeseth et al., 2015) have been used to address these issues. Coagulation - flocculation is most commonly used for the separation or purification of wastewater, which is an eco-friendly, cost-effective, and easy to operate procedure (Dao et al., 2016). Practically by adding coagulants to the wastewater, small particles or colloids are destabilized in coagulation processes. This destabilization of colloidal suspensions is achieved through double-layer compression, charge neutralization, entrapment, and the bridging of colloidal particles (Chee et al., 2016). Following the coagulation process, flocculation occurs, in which destabilized particles coalesce into bigger flocs that can be successfully removed by sedimentation (Yang et al., 2016).

Coagulants are mainly categorized into two classes, i.e., inorganic and organic coagulants. The inorganic coagulants contain multivalent metal ions. Examples of inorganic coagulants include iron (III) chloride, iron (II) sulfate, slaked lime, potassium aluminium sulfate, polysalts, and so on (Pariatamby and Kee, 2016). Ideally, they are added to the water suspension at high turbulence to cause the formation of precipitation or floc that can help entrap the particles and which can be separated through filtration or sedimentation. The generation of excessive non-biodegradable sludge is the major issue of using inorganic coagulants for wastewater treatment (Maurya and Daverey, 2018). Synthetic coagulants as well as natural coagulants are included in organic coagulants. The synthetic polymers contain acrylic resin, i.e., poly (2-propenamide) and its derivatives, including acrylamide acrylic amide and poly (ethylene imine). These chemically synthesized polymers cause severe neurotoxicity effects (Omyebuchi et al., 2020). The natural coagulant is also known as a bio-coagulant. Therefore, the advance of natural polymeric organic coagulants, such as chitosan, bio-based natural coagulants such as starch and cellulose has been widely promoted as the coagulation-flocculation technique. Due to bio-based natural polyelectrolytes, coagulants are entirely free of toxicity, biodegradable, and locally accessible (Gandiwa et al., 2020).

The effectiveness of different plant extracts on removing turbidity in wastewater has been examined by researchers. Plants including rice, wheat, groundnut, beans, and *M. oleifera* are considered the most common natural bio-coagulants used for treating wastewater (Shan et al., 2017; Zaid et al., 2019). In addition, plant-based natural coagulants combine efficiency, inexpensive capital and operating costs, simplicity of storage, supply reliability, sludge concerns, and compatibility with other treatment processes (Saleem and Bachmann, 2019). Regardless, operational conditions, including mixing parameters, coagulant dose, pH, solubility, and control strategy, might influence the external generation of a certain amount of sludge. Therefore, the green environmental concept emphasises producing a sustainable, alternative bio-coagulant from renewable sources (Keogh et al., 2017).

Earlier studies have confirmed that *M. oleifera* seed has been reported as a coagulating agent for purification. The powder obtained from *M. oleifera* seeds proved an effective alternative bio-coagulant. The powdered seed of *M. oleifera* has coagulating properties that have been implemented for various aspects of wastewater treatment, such as turbidity, alkalinity, solids (total dissolved solids and total solids), and hardness (Arnoldsson et al., 2008). *M. oleifera* coagulant (MOC) was used in raw and tannery industrial effluent waters for turbidity reduction in the range of 80–90% (Mageshkumar and Karthikeyan, 2016).

Using *M. oleifera* seed as a coagulant relies on water-soluble proteins containing a positive charge called the MOCP (*Moringa Oleifera* Cationic Protein). When this bio-coagulant is introduced to sewage wastewater, electrostatic attraction occurs, and cationic protein acts as a magnet. Water is magnetized by this cationic protein, which attracts anions. Due to this electrostatic condition, the proteins attract impurities in water and form flocs through the aggregation of particles. Then flocs are easy to remove by settling or filtration (Alakaparamil, 2020).

Our recent efforts have been focused on the coagulation process with *M. oleifera* seed powder (MOSP) for sewage water treatment. The main objectives of the present work are to investigate the coagulation potential of MOSP to remove impurities from municipal sewage water. This study also investigated the performance of various coagulants (natural, HCl, NaOH, and NaCl treated MOSP) to identify effective bio-coagulants to treat sewage water. Moreover, the performance of coagulating agents has been evaluated by measuring the electrical conductivity, pH, turbidity, biological oxygen demand (BOD), chemical oxygen demand (COD), and total solids (dissolved and suspended). The relationship between turbidity removals and temporal variation has also been investigated by pseudo-first and second-order kinetics models. The current study also aims to determine whether the prepared coagulants meet the criterion defined by WHO by evaluating the critical coagulation rate.

## 2 Materials and methods

### 2.1 Chemicals and reagents

All the chemicals, including concentrated hydrochloric acid (HCl), sodium hydroxide (NaOH), sodium chloride (NaCl), and reagents, were standard analytical grade and purchased from Hi-media, India, to maintain the expected accuracy and precision. In this experiment, an Indian 700 Eutech pH meter was used to evaluate the pH level of the water solution. The ionic conductivity of the treated solution was determined by a digital conductivity meter (306 Systronics, India). The turbidity was measured with a turbidity meter (135 Systronics, India), and the units were noted in NTU (Nephelometric Turbidity Unit). The entire experiment was carried out in distilled water at room temperature.

### 2.2 Collection of samples

The samples were collected from the municipal sewage canal at latitude: 21°30′46.70″N and longitude: 70°27′5.51″E in Junagadh, Gujarat, India. The sewage water sample has been collected in an air-tight amber plastic container. Before collection, the amber plastic container was cleaned with alcohol, rinsed 2–3 times with sterile distilled water, and dried at 80 °C for 30 min. The plastic container was flushed with sewage wastewater 1–2 times, followed by a collected sample (5L). The water sample was stored at 4^0C^ for further analysis (APHA et al., 2017; Maria et al., 2022).

Fresh seeds of *M. olifera* have been collected from the Girnar mountain region of Junagadh. The matured green, partially dried, and thoroughly dried pods have been collected from the *M. olifera* tree. All the collected pods are cleaned with distilled water, followed by sundry for seven days after being harvested. At room temperature, the harvested seeds had been dehusked and dried for 24 h. The dried seed sample was pulverized using a domestic grinder. The pulverized material was filtered through a 10 mesh (>2 mm) sieve to remove debris. It's important to note that the seed powder is highly attracted to moisture both before and after grinding. As a result, it is essential to dry the seeds powder at 40 °C for 10 min to reduce moisture content. After that, the seed powder was placed in desiccators to eliminate moisture, and the seed powder sample was placed in a clean, airtight polythene bag. Prepared seed powder is kept at 4 °C to avoid contamination (Gaikwad and Munavalli, 2019; Zaid et al., 2019).

### 2.3 Preparation of bio-coagulants

The *Moringa oleifera* was predominantly found in the Gujarat state and was cultivated in many districts due to its high nutritional and medicinal values. Therefore, the research study aims to prepare biocoagulants from *Moringa oleifera* seeds to treat sewage wastewater. Various treatments have been proposed to activate bio-coagulants, including the use of acid, base, and salt hydrolyzing agents to chemically activate *Moringa oleifera* seed powder (MOSP). For acidic and basic activation, 0.05M HCl and 0.05M NaOH were used. Moreover, 0.5M NaCl was used for salt activation. To perform a comparative analysis, MOSP has been activated with distilled water. The various dosages of MOSP (viz., 0.1, 0.5, 1.0, 1.5, and 2.0 ​g ​m) have been used to treat sewage wastewater (data not shown). Amongst them, 1 ​g ​m of MOSP concentration was found to be suitable for sewage wastewater treatment. Because 1 ​g ​m of MOSP helps to remove the total solids, pH, and turbidity from wastewater. As a result, 1.0 ​g ​m of MOSP was utilized for further study.

Furthermore, 100ml of hydrolyzing solution such as distilled water and chemical solutions (i.e., 0.05M HCl, 0.05M NaOH, and 0.5M NaCl) were taken and added to 1 ​g ​m of MOSP into the solution. These MOSP and hydrolyzing agents contain solutions mixed with vigorous stirring at 180 rpm for 60 min. After the stirring, the mixture was filtered through Whatman filter paper 1. The remaining residues were collected and referred to as water (natural), HCl, NaOH, and NaCl treated MOSP residues. Recovered residues were oven-dried at 50 °C for 24 h and then kept in a desiccator to remove moisture content. These treated MOSPs are water (natural), HCl, NaOH and NaCl treated bio-coagulants. The treated recovered material has been used as bio-coagulant material for further wastewater treatment (Naruka et al., 2021; Sivakumar et al., 2020).

### 2.4 Bio-coagulant experiments

The bio-coagulant experiment was followed to evaluate the efficiency of MOSP with and without chemical treatments as coagulant aid (Gaikwad and Munavalli, 2019). The coagulant experiments were performed by adding 1 ​g ​m of natural, HCl, NaOH, and NaCl treated MOSP in individual beakers containing 500 mL of filtered (without larger detritus) sewage wastewater (Maurya and Daverey, 2018). Following the addition of the coagulant doses, the mixtures were shaken rapidly for 5 min at 150 rpm, followed by slowly for 30 min at 50 rpm, and finally at 20 rpm for 10 min. Slow mixing was continued until large flocs formed and then settled at room temperature. The samples were collected without disturbing the stable particle and filtered for further investigation (Manisha and Sudarsan, 2017). The suspensions of all treatments were incubated at room temperature in dark conditions up to 14^th^ day, and the same procedure has repeated at the interval of 48 h up to the 14^th^ day (Anjaneyulu et al., 2005; Naruka et al., 2021). Natural, HCl, NaOH and NaCl treated MOSP were used for Physico-chemical analysis to determine the coagulation effect on sewage wastewater treatment. To measure turbidity is one of the fundamental factors to evaluate the turbidity in NTU, which is indicated muddiness or haziness present in the sample. The bubble formation during the treatment had to be evacuated by gently shaking during the monitoring. According to Tchobanoglous et al. (2003), residual turbidity measured in NTUs was converted to mg L^−1^ by multiplying with 2.3.

### 2.5 Environmental parameters of water suspension

The effectiveness of biobased coagulating materials has been determined by monitoring the environmental parameters, including turbidity, conductivity, pH, and suspended solids of water suspension. After collecting the sample, immediately assessed the pH level and electrical conductivity. Furthermore, the total suspended solids (TSS) were examined to clarify suspended particles' dissolving ability in water samples. TSS was determined by a gravimetric measurement of the residue dried to a consistent weight for at least 1 h at temperatures ranging from 103 °C to 105 °C. TSS refers to waterborne particles that exceed 2 microns in size. The filtration process removes these particles (Feng et al., 2017; Naruka et al., 2021).

Moreover, total dissolved solids (TDS) are measured to dissolve organic and inorganic substances in water suspensions. The TDS is calculated by evaluating the weights of total solids before and after drying the sample. The sample was dried in a hot air oven at 105^0C^ until the water evaporated entirely (Naruka et al., 2021). The biological oxygen demand (BOD) of water was measured by Winkler's method. BOD measures the amount of oxygen required to decompose organic matter in wastewater. The chemical oxygen demand (COD) measured the water quality by the closed reflux method. The environmental parameters, including TSS, TDS, BOD, and COD, were calculated by the American Public Health Association (APHA et al., 2017) and suggest precautions are being taken to avoid contamination.

### 2.6 Coagulation kinetic modeling

The Brownian motion of suspended particles directs the early stages of coagulation. The Brownian motion of the particles because of the thermal motion of water molecules is random, and this random motion causes the particles to collide. When the particles have been completely destabilized, flocculation occurs when they collide, allowing small particles to aggregate into larger particles. The mass of solid particles in the water remains unchanged (Sun et al., 2019). The collision of particles caused by Brownian motion results in “isotropic flocculation,” which happens during the mixing stage and is also known as “coagulation”.

Furthermore, the kinetics controlled the floccule formation rate and facilitated the accomplishment of the crucial period before the floccules destabilized. This flocculation is known as “co-flocculation”. It is an essential part of the coagulation process (Sun et al., 2019). The rate Eq. (1) expresses the coagulation kinetics modelling by calculating and reducing turbidity.

(1) $$-\frac{dC}{dt}={\text{kC}}^{\text{n}}$$

Where *C* is the final turbidity level in mg L^−1^, *t* is the coagulation time (min), and *k* is the *n*th order rate constant. ‘n’ is the kinetic order of the coagulation process and their theoretical values are in the range (1 ≤ n ≤ 2) as described by Naruka et al. (2021). The minus sign represents the reducing turbidity level with an increase in time. If coagulation follows the first order kinetics where *n* = 1, then the rate equation is;

(2) $$-\frac{dC}{dt}={\text{k}}_{1}{\text{C}}^{1}$$

The integration of Eq. (2) becomes:

(3) $$ln\left(\frac{Co}{C}\right)={\text{k}}_{1}\;1$$

Where *C*_*0*_ is the initial turbidity level in mg L^−1^, *C* is the final turbidity level at time (*t*), and *k*_*1*_ is the pseudo-first order kinetic constant (1/min) showed in Eq. (3).

If the coagulation follows the second order kinetics n = 2 described by Nwabanne and Obi (2019), and the equation is;

(4) $$-\frac{dC}{dt}={\text{k}}_{2}{\text{C}}^{2}$$

Eq. (4) integrates with the boundary conditions initial (*t = 0, C = C*_*0*_) and final (*t = t, C = C*).

(5) $$\frac{1}{\text{C}}={\text{k}}_{2}\;\text{t}+\frac{1}{{\text{C}}_{\text{0}}}$$

Rearranging Eq. (5) to calculate the second order kinetic (k_2_) rate using Eq. (6),

(6) $${\text{k}}_{2}=\frac{\frac{1}{\text{C}}-\frac{1}{{\text{C}}_{\text{0}}}}{t}$$

Where k_2_ is the pseudo-second order coagulation rate constant (L mg^−1^ min) and the aggregations of coagulating particles generally follow the second order kinetics model.

The relationship between friction factor (*β*, m^3^ kg^−1^ s) and *n*th order coagulation rate constants calculated by Eq. (7),

(7) β = 2k

Brownian diffusion coefficient (*D*, kg^2^m^−1^ s) is determined by the Boltzmann constant (*k*_*B*_), temperature (K) and friction factor (Okey-Onyesolu et al., 2020). The diffusion coefficient is correlated by Eq. (8),

(8) *D* = k_B_T/ *β*

Where *k*_*B*_ = 1.3806452 × 10 ^−23^ $\left(\frac{\text{m}2\text{kg}}{\text{s}2\text{K}}\right)$

Half-life period (t_1/2_) is the time required for the initial level of turbidity to reduce to one-half of the original value (Naruka et al., 2021). The half-life period for the pseudo - first order kinetic is calculated by Eq. (9);

(9) $${\text{t}}_{1/2}=\frac{ln2}{k1}$$

The half-life period for the pseudo - second order kinetic is calculated as per Eq. (10);

(10) $${\text{t}}_{1/2}=\frac{1}{{\text{k}}_{2}{\text{C}}_{\text{0}}}$$

### 2.7 Characterization of coagulants

Structural and morphological identification of natural and chemically treated MOSP were analyzed by scanning electron microscopy (SEM). Moreover, Energy dispersive X-ray (EDAX) was used to examine the elemental composition of the studied materials. Furthermore, to observe the functional group of the study materials, they were characterized by Fourier transform infrared spectroscopy (FT-IR) spectroscopy analysis (IR-Spirit, Shimadzu, Japan).

## 3 Results and discussion

### 3.1 Selection and preparation of plant-based coagulants

The mature fruits of the *Moringa oleifera*, commonly called the drumstick tree. The mature fruits (pods) were collected and assorted. *Moringa oleifera* is a tropical plant of the Moringaceae family (Vieira et al., 2010). The seed's wet biomass was approximately 93 ​g ​m with testa, and it was then washed with sterile distilled water and dried at 80 °C for 5 days. The quantified the dried seed biomass, i.e., 67.3 g.

Furthermore, the seed was pulverized by a grinder and used as a plant-based coagulant to treat municipal sewage water. The chemical modification of plant-derived material is well known for improving wastewater's biological and physico-chemical properties; hence, it is anticipated to open a new avenue for wastewater treatment (Maurya and Daverey, 2018). The surface modification of coagulants controls their structural properties, including functional groups, and enhances the buffering capacity of water. Hence, the prepared MOSP coagulants improve waste materials' coagulant capacity and flocculation properties. Thus, the modification has facilitated the removal of organic pollutants and is a cost-effective approach for wastewater treatments (Teh et al., 2016; Yang et al., 2016). Thus, it can be commercialized in the future due to its richness and other ecofriendly value-added properties.

### 3.2 Physico-chemical parameters of water

#### 3.2.1 Effect on pH

**Figure 1:**
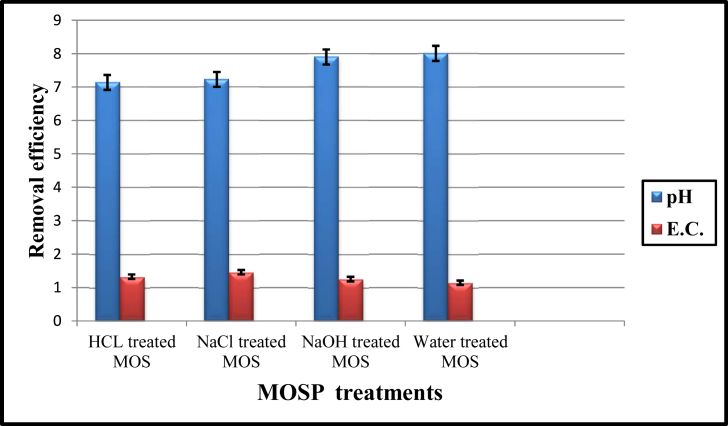
PH and Electrical conductivity (μScm-1) removal efficiency after MOSP bio coagulants treatments.

Coagulation activity is heavily influenced by the pH (proton concentration) of the solution (Hendrawati et al., 2016). The pH of municipal sewage water was recorded to be alkaline as 8.03 ± 0.02. The addition of activated bio-coagulant to water suspension and mixed the content in controlled conditions for 30 min. Then, the content was allowed to settle down at room temperature, and then the pH change was measured and recorded (Figure 1). Activated MOSPs showed a drastic significant pH reduction and decreased from alkaline to near neutral pH. The maximum pH reduction was observed in HCl treated MOSP coagulants and was recorded to be 7.14, followed by NaCl, NaOH and natural, treated MOSP which were found to be 7.29, 7.9, and 8.01, respectively. The initial and treated water by all MOSPs were within the range, i.e., 6.5 to 8.5 (WHO, 2017). Therefore, it is indicated that the HCl treated MOSP proved to be an effective coagulant for reducing pH. According to Shahzad et al. (2014), the presence of low molecular weight proteins in activated MOSPs resulted in a lowered pH of sewage water. These proteins carry a positive charge and attract negatively charged particles such as toxic materials. As a result of the reaction, free hydrogen ions (H+) are generated from the hydrolysis reaction, which causes the pH to decrease when coagulants are added to the solution (Hendrawati et al., 2016). Therefore, the study revealed that the chemically treated MOSP had enhanced the coagulation-flocculation due to destabilizing negatively charged particulates. Adding 1 ​g ​m of dose as a coagulating agent in 500 mL of sewage water was found to be adequate to reduce the pH and observed to be near to neutral, which subsequently reduces the turbidity of water by *Moring oleifera.* As a result, these biomaterials could be used as bio-coagulants to help maintain a neutral pH in wastewater, one of the most promising characteristics in terms of portability (Varsha et al., 2010).

#### 3.2.2 Effect on electrical conductivity

The effect of electrical conductivity (EC) on the coagulation activity of sewage water was determined, which was found to be 1302 μS cm-1 reported in untreated water. In addition, after the treatments of various coagulants, the EC was found to be maximum in NaCl, HCl, and NaOH treated MOSP, and the minimum was found in natural MOSP compared to untreated water reflected in Figure 1. The EC in untreated water may increase due to the water-soluble seed proteins that attract the negative charge particles in the aqueous water solution. Because MOSP coagulants are pH reducers at high alkaline conditions, it may take longer to settle the particles and negative charges to be removed from the sewage water (Naruka et al., 2021). Hence, the electrical conductivity was found to be maximum in the coagulant treated water compared to untreated water. The positive charge coagulants were subjected to neutralizing sewage water's electrical charge, which resulted in negative turbidity. Moreover, the positive charge of natural MOSP was anticipated to remove the negative charge turbidity, subjected to reducing sewage water's pH. Another reason is that plant contain so many minerals and inorganic compounds, which dissolve in water and cause high conductivity in water (Figure 1). The addition of water and NaOH activated coagulant MOSPs may result in the dispersion of some mineral ions and inorganic compounds into a floc, which will then be precipitated and separated from the water suspension, which may result in a slight reduction of electrical conductivity. The reaction between water and charged metals increased water ionic conductivity in the presence of salt and acid-activated MOSP coagulants. It also reacts with inorganic compounds that dissociate in water, contributing to water's ability to conduct substantial electric currents. Hence, water's ionic conductivity or electrical current ability depends on the concentration of charged ions in water. In the coagulation process, *M. oleifera* seeds have little effect on conductivity (Hendrawati et al., 2016).

#### 3.2.3 Effect on TSS and TDS

**Figure 2:**
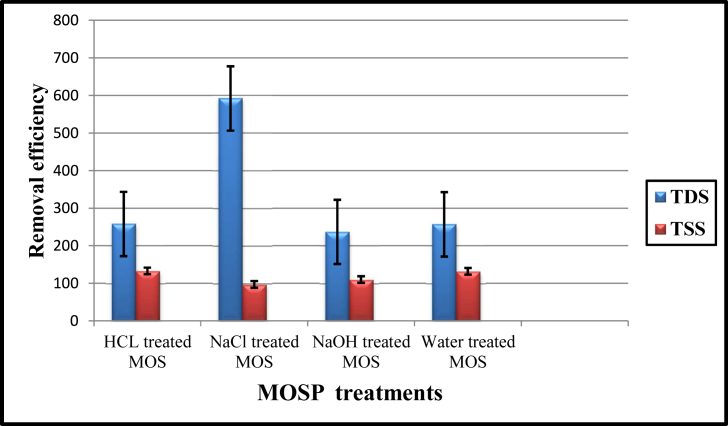
Total dissolved solids (TDS) and total suspended solids (TSS) reduction capacity in mg/L after MOSP bio coagulants treatments.

Total Dissolved Solids and total suspended solids are the total amounts of movable charged ions dissolved in a given volume of water, including minerals, salts, and metals. The amount of solid content (TSS and TDS) is measured in milligram per unit volume of water (mg L^−1^). The quality of wastewater purification systems is directly related to TSS and TDS (Sunita and Sonal, 2014). The impact of TSS and TDS was frequently investigated before and after the coagulant processes. The concentration of TSS ranged from 97 to 133 mg L^−1^, and the maximum reduction of solid contents was reported to be 97 mg L^−1^ in NaCl treated coagulants. It indicates that the destabilization of suspended solids at optimum pH and optimum time for rapid mixing is often achieved (Figure 2) (Sorokhaibam et al., 2014). Therefore, the coagulation effectively formed the floccules or precipitates of the given sample (Jeyasundar et al., 2020). Coagulation or destabilization of a colloidal suspension resulted in the agglomeration of small particles by physical and chemical processes that were settled down in the medium and filtered. Therefore, the coagulation dose was adequate to reduce the solid content of the water suspension (Saritha et al., 2017). The TDS concentration ranged from 237 to 954 mg L^−1^. As per the WHO standard, less than 600 mg L^−1^ is generally considered to be suitable, and if the concentration is more significant than 1000 mg L^−1^ suggested that the water is unpalatable. It may require appropriate treatment before use (WHO, 2017). During the experiment, the initial TDS was found to be a maximum of 954 mg L^−1^ in untreated water. After the treatment by various coagulants (viz. natural, HCl, NaCl and NaOH treated MOSP), it was resulted to have a reduction of TDS level up to 76% (i.e. 954 to 237 mg L^−1^) (Figure 2). The maximum removal of TDS was 237 mg L^−1^ recorded in NaOH treated coagulants, which proved to be a better coagulant for sewage water treatment. Therefore, the present study suggests that the MOSP coagulants efficiently remove solid content from sewage water, especially TSS and TDS. Activated MOSP has a cationic charged protein that binds anionic charges on liquid waste through floc sedimentation, leading to a decrease in total dissolved solids and total suspended solids levels. This statement is strengthened by Suleyman and Lilian (1995). Thus, the study revealed that the NaOH MOSP coagulants are effective against sewage wastewater. Overall, the available MOSP exhibited potential coagulation activity to remove solid contents from the sewage wastewater.

#### 3.2.4 Effect on oxygen demand (BOD and COD)

**Figure 3:**
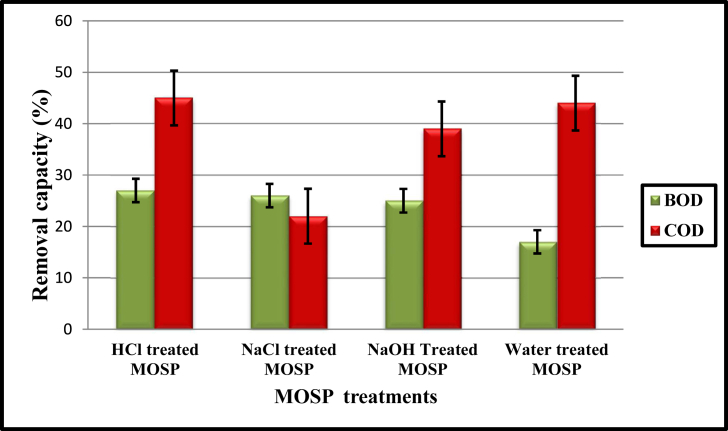
BOD and COD removal capacity after MOSP bio coagulants treatments.

During high water pollution, bacteria present in sewage water break down and feed on complex organic matter into simple compounds such as carbon dioxide and water, leading to bacteria multiplication. The investigation of coagulants on the reduction of BOD and COD by applying the various chemically treated MOSP coagulants in the sewage wastewater. The effect of the coagulants on the removal of BOD and COD is revealed in Figure 3. The reduction of BOD fluctuated from 27 to 17 %, and the maximum was found to be 27% in HCl treated MOSP, followed by 26%, 25%, and 17% were noted in NaCl, NaOH, and least in natural MOSP coagulants, respectively. The removal of BOD is due to reducing microbial growth during the treatments with MOSP coagulants. Therefore, the present study revealed that the MOSP could be used as an alternative for treating municipal sewage water compared to chemical coagulants (Igwegbe et al., 2021). The initial concentration of BOD in an untreated sample was 512 mg L^−1^. After the treatment with coagulants, especially treated with HCl, the BOD was reduced and recorded as 27% (Figure 3).

In the environment, chemical oxygen demand (COD) with an excessive amount is considered hazardous. Therefore, the COD removal efficiency was monitored during the experiments, and the data is shown in Figure 3. The removal of COD ranging from 45 to 22% was recorded in MOSP coagulants. The maximum reduction of COD was reported in HCl treated coagulants, followed by 44, 39, and 22% reduction in natural, NaOH, and NaCl treated MOSP coagulants, respectively. The results showed that the overall treatments with bio-coagulant extracted from *M. oleifera* seed, COD increased when the MOSP coagulants were changed. On the other hand, the COD value is directly proportional to the chemical treatment used to prepare bio-coagulants. Thus, the results shown in Figure 3 represent the percentage removal of COD in various chemically treated coagulants. According to the Alam and Sirajul (2017) results the maximum removal of BOD and COD was found to be 22 and 38% in industrial wastewater using *N. officinale*, respectively. The % removal efficiency were found to be maximum in the present study as compare to the previous studies (Table 2).

Moreover, the natural coagulants also showed better results than the chemically treated coagulant due to *M. oleifera*, which had established the higher coagulants capacity of organic matter. A decrease in COD values in chemically treated MOSP may be due to the protein and natural organic compounds present in the seeds (Nhut et al., 2020). The possible mechanisms by which the organic matter from sewage water samples has been removed by bio-coagulant from *M. oleifera* are dominated by adsorption and charge neutralization (Nonfodji et al., 2020). In addition, COD removal was combined with a decrease in turbidity in the water. After the COD removal, coagulants were one of the major functions for the coagulation activities. The coagulants prepared from the *M. oleifera* seed primary mechanism of coagulants and interparticle bonds play a significant role in the coagulation process (Teh et al., 2016) due to the long-chain molecular structure that allow for the coagulation of organic matter from the sewage water. As a result, MOSPs have shown tremendous efficiency for reducing dissolved oxygen (DO, BOD, COD) in sewage wastewater (Nhut et al., 2020). Thus, the present study revealed that the MOSP is an alternative, cheaper, and eco-friendly coagulant than chemical coagulants (i.e., aluminium sulfate) to treat environmental pollutants such as municipal sewage water. Wang et al. (2014) reported that the use of *M. oleifera* in wastewater treatments helps to reduce the harmful effects on human health and the environment. Hence, the MOSP is seen to be an effective bio-coagulant that has significant efficiencies in treating organic pollutants.

### 3.3 Turbidity kinetics

The optimum bio-coagulant doses (1 ​g ​m) have been used to treat sewage water, which resulted in the removal of turbidity from the water. An initial turbidity of 20.6 NTU was reported in untreated water. The maximum reduction of wastewater turbidity from 20.6 to 1.3 NTU indicated almost 84.48–93.68% removal was recorded in various bio-coagulant treatments. The higher turbidity removal was 1.3 NTU recorded in HCL treated MOSP, followed by NaOH (1.9 NTU), NaCl (2.3 NTU), and least was found in natural (3.2 NTU) MOSP coagulants. Various studies reported that the MOSP could reduce the turbidity, especially using the seed of *M. oleifera* as bio-coagulants*. M. oleifera* seeds have been reported to contain active bio-coagulation compounds and reduce high turbidity (Madrona et al., 2010; Hamid et al., 2014; Gaikwad and Munavalli, 2019). The MOSP ability has been explained by reducing the turbidity of sewage water by coagulating the neutralizing negative charged particles of the colloidal. Therefore, usage of activated MOSPs for sewage wastewater treatment would be a relatively promising, cost-effective, and eco-friendly technology (Nhut et al., 2020).

**Figure 4:**
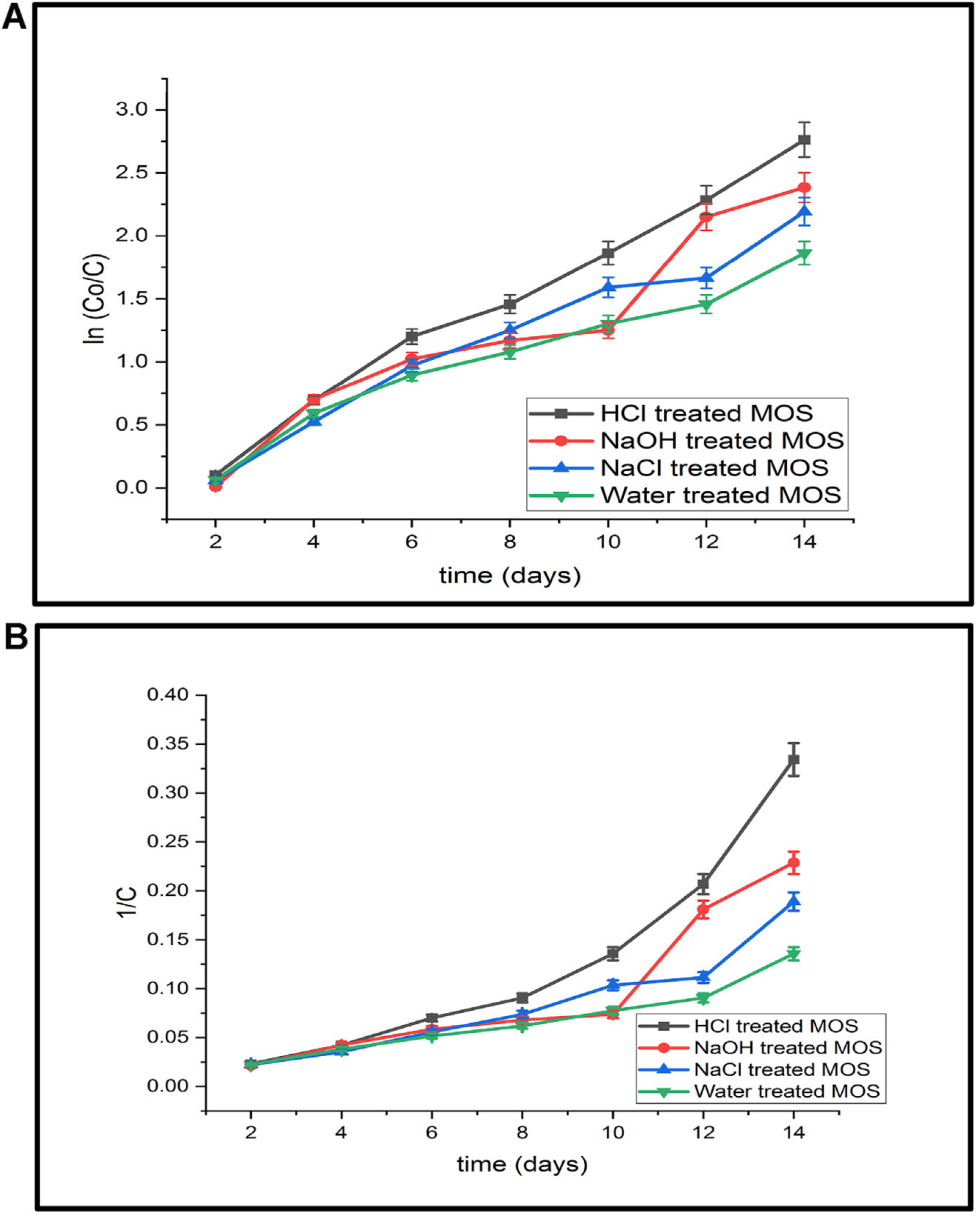
A. pseudo-first-order kinetic model of different functionlized MOSPs during biocoagulation in sewage wastewater. B. pseudo second-order kinetic model of different functionlized MOSPs during biocoagulation in sewage wastewater.

The coagulation kinetics revealed that the turbidity decreases in the water sample with an increase in time. The variation of residual turbidity concentration with time in various AMCs was investigated to better interpret coagulation kinetics. The preliminary studies reflect that the time taken to attain equilibrium is 30 min for a coagulation process. We used a first-order process rate equation to test the experimental data and thus to explain the coagulation kinetics. According to the R^2^ and RMSE profiles, the value demonstrated that the observed data fit with the first-order kinetics model (Table 1). Therefore, the rate of equations for first-order was considered. As shown on the plot (Figure 4A), the Y-axis intercept is in accordance with the first-order kinetic equation. It should be noted that the correlation coefficient (R^2^) found from first and second-order plots revealed the linearity of the plot (Table 1). The results showed that turbidity reduction is relatively faster in HCl treated MOSP than in other bio-coagulants (Fig.4A-B). Generally, WHO (2017) recommends consumers drink water with turbidity less than 5 NTU, although it may vary depending on the type of water and the conditions. The mathematical formula is represented in terms of critical coagulation rate constant (k_c_) could be introduced according to Eq. (11);

(11) 1/C = k_c_t + t/C_0_

Rearranging equation (11) for calculating the value of k_c_;

(12) $${\text{k}}_{\text{c}}=\frac{\frac{1}{\text{C}}–\frac{1}{{\text{C}}_{0}}}{t}$$

Where, C is the accepted upper limit of water turbidity (≤5 NTU) as suggested by WHO (2017), C_0_ is the initial turbidity concentration of waters tested (sewage water, 20.6 NTU), and t is the predetermined settling time of 30 min. The performance of each bio-coagulant (MOSP) over sewage water was assessed in terms of kc as per Eq. (12) with the performance criterion being k2≥ k_c_ for the sewage water (Al-Sameraiy, 2017). The sewage wastewater had a kc value of 0.024 (1/NTU min), lower than the k2 value for municipal sewage water.

**Table 1:** Pseudo-first and second order kinetic constants during the bio-coagulation of sewage water through chemically activated MOSP.

	Kinetic variable	HCL treated MOSP	NaCl treated MOSP	NaOH treated MOSP	Water treated MOSP
**First order**	*k*_*1*_ (L min^−1^)	0.19735	0.170246	0.156599	0.13301
	*R*^*2*^	**0.99201**	0.93905	0.97652	0.96723
	*RMSE*	**0.05**	0.13	0.07	0.05
	*β* (m^3^/kg s)	0.3947	0.3404	0.3131	0.2660
	*D* (kg^2^/m s)	0.97 × 10^−21^	1.13 × 10^−21^	1.23 × 10^−21^	1.45 × 10^−21^
	*t*_*1/2*_ (min)	3.51	4.07	4.42	5.21
**Second order**	*k*_*2*_ (L min^−1^)	0.022	0.014	0.011	0.008
	*R*^*2*^	0.875	0.829	0.909	0.935
	*RMSE*	1.68	0.02	0.01	0.008
	*β* (m^3^/kg s)	0.044	0.0296	0.023	0.016
	*D* (kg^2^/m s)	0.86 × 10^−20^	1.30 × 10^−20^	1.61 × 10^−20^	2.35 × 10^−20^
	*t*_*1/2*_ (min)	0.94	1.42	1.75	2.57

**Table 2:** Comparison of BOD and COD removal efficiency (%) of prepared MOSPs with other coagulants.

No.	Coagulants	Water	BOD	COD	References
1	*N. officinale*	Industrial water	22	38	Alam and Sirajul, 2017
2	*C. procera*	Dairy water	6	15	Nawash et al. (2014)
3	*H. verticillata*	Domestic water	26	25	Chonyitree et al. (2021)
4	*A. indica*	Textile water	27	43	Mohan et al. (2019)
5	*W. lettuce*	Sewage water	33	26	Mumtaz et al. (2014)
6	*M. oliefera*	Domestic water	17	12	Kamoru et al. (2017)
7	*M. oliefera* (HCl treated)	Sewage water	27	45	Present study

**Figure 5:**
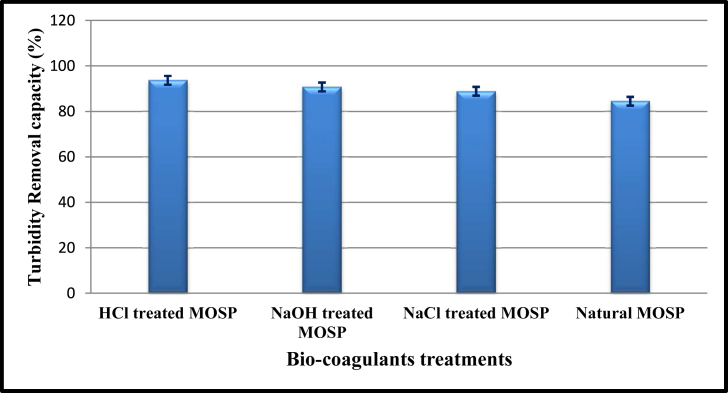
Comparison of turbidity removal in various functionlizedMOSPs bio-coagulants during sewage water treatments.

Furthermore, Table 1 showed that the t_1/2_ varied depending on the change in the MOSP bio-coagulants used. In the present study, the first order coagulation process has a more significant half-life than the second-order coagulation process. By comparing the model, the root mean square error (RSME) value of second-order kinetics was lower in all MOSP coagulants than the first-order kinetics, except for HCl treated water (Table 1). In every possible aspect, the second-order kinetics model proved to be most suitable and superior over the first-order kinetics constant. Figure 5 shows the turbidity removal capacity using the different bio-coagulating agents. The highest removal efficiency was 93.68% in HCl treated MOSP, and the least was recorded in natural MOSP i.e., 84.46% (Figure 5). Every coagulant resulted in more than 84% removal capacity with *M. oleifera* seed compared to the other chemical coagulants, viz., potassium aluminium sulfate and iron (II) chloride (Mageshkumar and Karthikeyan, 2016). Hence, this recent study suggested the MOSP could remove the turbidity very efficiently, and water could be considered within the permissible limits (WHO, 2017).

### 3.4 Scanning electron microscope (SEM) analysis

Scanning electron microscope (SEM) analysis determines the high resolution of coagulant surface images for visualization and characterization of the surface of particles. The SEM images of MOSPs before and after treatment are observed in Fig. S1 (A–E). The raw material was also being analyzed. Fig. S1 (A) separate fine particles were observed due to non–coagulation process, and aggregation has not appeared. There is a possible exchange of oppositely charged ions when the MOSP is treated with a HCL solution, leading to the formation of double-layer particles. The results in generating a second layer of particles, as seen in Fig. S1 (B), it favours particle aggregation once again. Fig. S1 (C) displays many particles aggregating into more significant, thicker, and easily aggregates, characteristic of the coagulation-flocculation process. On the other hand, the molecular weight and functional groups (Fig. S1-D), showed a highly heterogenic surface. It resembles thicker and bigger flocs. Subsequently, Fig. S1 (E) indicated thicker, dense, and large particle accumulation and formed a film like larger flocs responsible for coagulation. The SEM images reveal a substantial difference in each bio-coagulant's topography before and after wastewater treatment. According to the SEM images, it is demonstrated that each coagulant efficiently accomplished the coagulation process.

### 3.5 Energy dispersive spectroscopic (EDAX) analysis

Energy-dispersive spectroscopic analysis is used to study the coagulation efficiency for investigating the presence of elements. The elemental weight percentage of the raw MOSP sample indicates the presence of molecules like oxygen, magnesium, sulfur, and potassium, which were exhibited in concentrations of 64.67, 1.28, 7.94, and 6.04 wt %, respectively (Fig. S2A). Fig. S2B determines the presence of oxygen, magnesium, sodium, potassium, silicon, chlorine, and calcium with changes in weight percentage of 43.06, 6.57, 16.1, 2.56, 6.17, 10.58, and 6.87 wt%, respectively, from HCl treated MOSP. Whereas, in NaCl treated, MOSP exhibits 46.19, 6.03, 2.99, 2.98, 33.03, 6.91, and 1.87wt% for the occurrence of oxygen, sodium, gallium, silicon, niobium, chlorine, and potassium elements (Fig. S2C). Fig. S2D determines the presence of molecules like oxygen, sodium, magnesium, silicon, chlorine, potassium, and calcium with a weight percentage of 45.13, 10.80, 5.24, 10.26, 5.67, 2.34, and 7.67, respectively, from the NaOH treated MOSP sample. Fig. S2E indicates that the natural MOSP sample exhibits 33.88, 14.39, 2.71, 1.79, 2.64, 9.59, 9.37, 1.37, and 3.66 wt% of oxygen, sodium, aluminium, sulphur, magnesium, silicon, chlorine, potassium, and calcium, respectively. In general, the presence of the elements, as mentioned earlier due to chemical treatment, may lead to particle destabilization through chemical reactions between coagulant and colloids, according to the EDAX analysis (Saritha et al., 2017). This is followed by the flocculation transport of destabilized particles that will cause a collision with floc for optimum turbidity. EDAX analysis proved that raw *M. oleifera* seed powder consists of only simple elements (oxygen, magnesium, sulfur, and potassium). After wastewater treatment with activated *M. oleifera* seed powder, some trace elements are present in the flocs of MOSPs. Hence, a comparative study of EDAX analysis between raw *Moringa* seeds and activated MOSPs showed that *Moringa* seeds have a prominent ion-exchange capacity in wastewater treatment during coagulation.

### 3.6 Fourier transforms infrared spectroscopy (FT-IR) analysis

Comprehensive characterization and functional group identification of the materials are quickly and effectively characterized by FTIR (Arulmathi et al., 2019). These functional groups are present in the floc produced due to the coagulation-flocculation process in wastewater (Katrina and Ta, 2015). A fundamental concept in infrared (IR) spectroscopy is that molecular or atomic vibrations in crystal lattices at particular wavelength that correspond to internal movement of atoms within molecular or atomic group structures (Radha et al., 2020). The spectrum observed for the floc produced by the treatment of activated *M. oleifera* seed powder. Fig. S3 (A) FT-IR spectrum showed natural MOSP peak range between 3000 cm−1–1000 cm−1. It was attributed to the presence of the carboxylic acid, amine, and imine groups due to the solid stretch of the O–H bond at 3296 cm−1, the vibration of the C–O bond at 1660 cm−1, a medium stretch of C–N at 1235 cm^−1^, and 1055 cm^−1^ double stretch of C = N bonds. The peak of the HCl-treated MOSP sample was shown in Fig. S3 (B) at 3500cm-1 to 660 cm-1. A Sharp peak shows at 3310 cm^−1^ due to O–H stretch; medium stretch represents the alkane functional group at 2700 cm^−1^ to 2900 cm^−1^. The peak at 1657 noticed that the medium stretch of the amine functional group is due to the vibration of C = N stretch. The peaks at 1233 cm-1, 1038 cm-1, and 668 cm-1 can be attributed to the sulfoxides and alkynes functional groups that are present in the strong vibration of the C–O, S = O, and C = C stretches. In Fig. S3(C), results of NaCl treated MOSP sample. A peak at 3296 cm^−1^ stands as an indication of an intense stretch of the C–H band. 2918 cm^−1^ stands for alkene due to the medium stretch of C–H. The peak at 1658 cm^−1^ determines the medium stretch of the O–H band. The peaks at 1233 cm^−1^ and 1034 cm^−1^ represent the strong single stretch of carbon, oxygen, and sulfoxide (S = O). A peak at 668 cm^−1^ indicates an alkenes functional group due to the strong stretch of the C = C band. Fig. S3 (D) NaOH treated MOS sample, and it showed a peak at 3287 cm^−1^ for an intense stretch of C–H band. 2918 cm^−1^ stands for a medium stretch of C–H. The Peak at 1628 cm^−1^ determines the stretch of O–H. A peak at 1228 cm^−1^exhibits the medium stretches of the C–N band and a peak at 872 cm^−1^ represent the strong vibration of carbon and oxygen stretch. A water treated MOS sample shows a different peak range than other samples (Fig. S3E). It offers a peak range between 3300 cm^−1^ to 660 cm^−1^. The carboxylic acid group is present at 3297 cm^−1^ due to O– H's strong, broad intermolecular bonding. The vast weak stretch of O–H is represented by a peak at 2929 cm-1 and 2851 cm-1. A Peak at 2360 cm^−1^ proved the vibration of the Si–H stretch. A medium stretch of alkene is present at 1653 cm^−1^. At 1457 cm^−1^, the vibration of the C–C stretch occurs. A substantial stretch of sulfur and oxygen is present at 1031 cm^−1^, while 668 cm^−1^ stands for the strong alkene C = C functional group. Hence, comparative analysis of before and after coagulation treatment indicates that Fig. S3 (A) consists of only 3–4(O–H, C–O, C–N, C=N) functional groups. In contrast, floc observed that some peaks are shifted after the coagulation process, and a new peak appears. It could be attributed to the reaction of cation-anion exchange capacity of the coagulants through electrostatic attraction (Bernard et al., 2021; Radha et al., 2020). Therefore, it is proved that the presence of these carboxyl, amine, or imine (O–H, C–O, C–N, C=N) polar functional groups on the surface may be responsible for the coagulation activity in wastewater.

## 4 Conclusion

• This coagulation-flocculation study reveals that the HCl activated MOSPs have greater efficiency in removing turbidity, BOD, COD, and solids from sewage wastewater.
• HCl treated MOSP has removed >84% of turbidity in the sample; it proved to be a better alternative for wastewater treatment.
• EDEX analysis revealed that the MOSP has a high removal efficiency of metallic ions. Moreover, FT-IR analysis also revealed that the MOSP contains carboxyl and amine groups (C–O, O–H, C=N, C=N), which facilitate the coagulation process.
• Overall, results achieved from the HCl treated MOSP suggests that they reveal a better alternative, toxic-free, cheaper bio-coagulant used in wastewater treatments.

## Declarations

### Author contribution statement

Vaishali Varsani: Conceived and designed the experiments; Performed the experiments; Wrote the paper.

Suhas Vyas: Analyzed and interpreted the data.

Dushyant Dudhagara: Analyzed and interpreted the data; Contributed reagents, materials, analysis tools or data.

Funding statement: This research did not receive any specific grant from funding agencies in the public, commercial, or not-for-profit sectors.

### Data availability statement

Data will be made available on request.

### Declaration of interests statement

The authors declare no conflict of interest.

### Additional information

No additional information is available for this paper.

## Appendix A Supplementary data

Supplementary file_V2.docx Supplementary file_V2.docx
