# Supplementary material for: Development of bio-based material from the Moringa oleifera and its bio-coagulation kinetic modeling–A sustainable approach to treat the wastewater
Source: Heliyon. 2022 Sep 1;8(9):e10447. doi: 10.1016/j.heliyon.2022.e10447 (PMC9468400; doi:10.1016/j.heliyon.2022.e10447)
Supplement: Supplementary file_V2.docx [file mmc1.docx]

**Supplementary materials**







**(B)**

**(A))**







**(D)**

**(C)**





**(E)**

**Fig. S1.** SEM analysis for morphological characterization of MOSP sample (A- raw MOSP material, B- HCl treated MOSP, C- NaCl treated MOSP, D – NaOH treated MOSP, E – water treated MOSP)


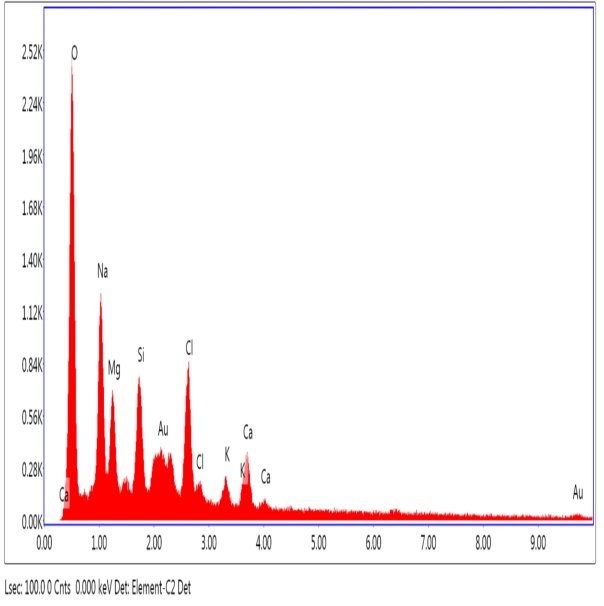

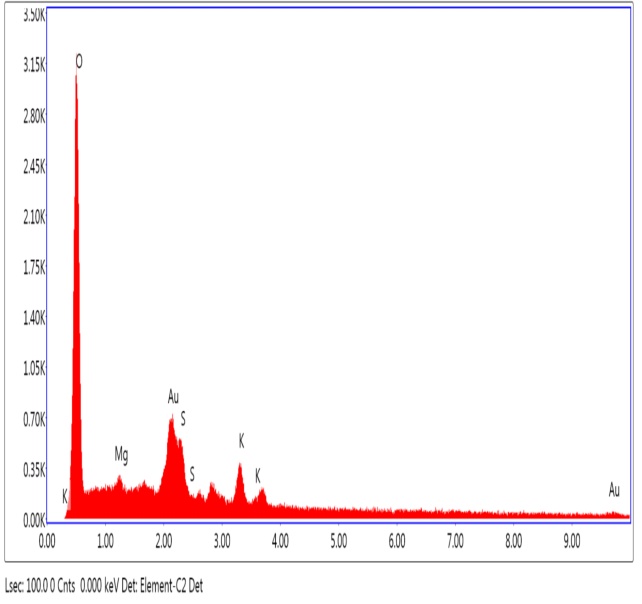


**(B))**

**(A))**


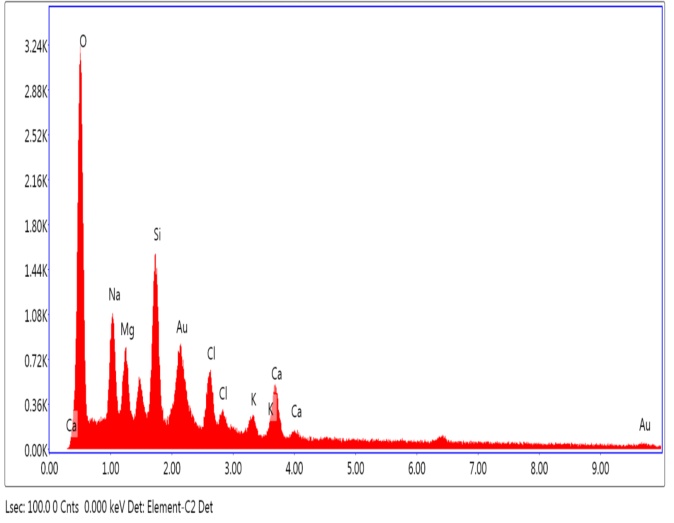


**(C))**


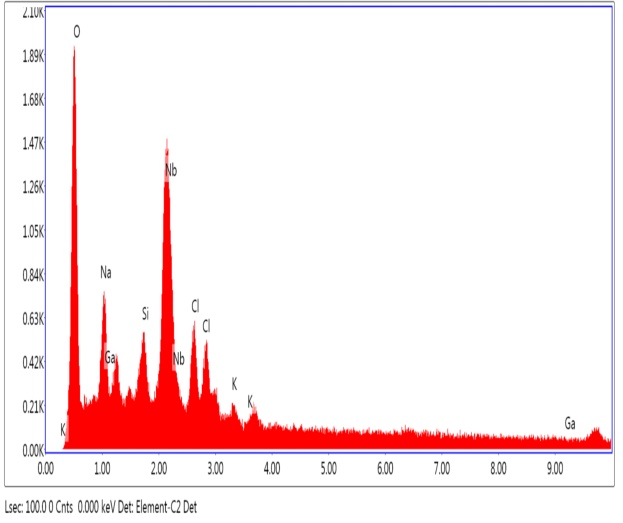


**(D))**


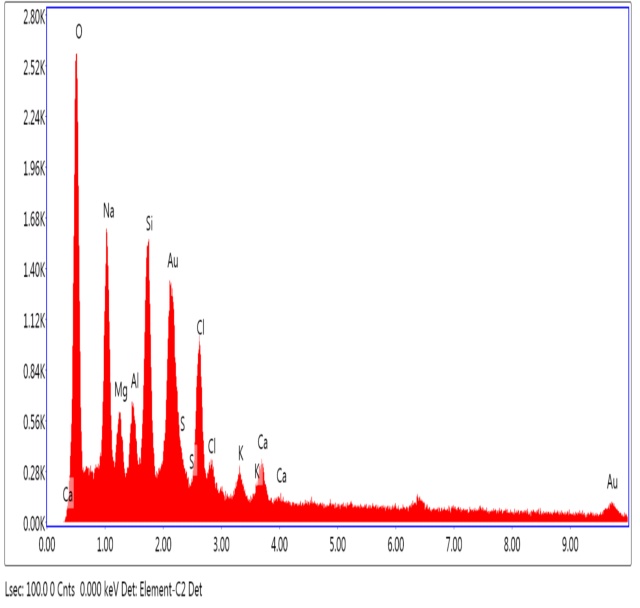


**(E))**

**Fig. S2** Energy dispersive spectroscopicanalysis for elemental composition of MOSP sample (A- raw MOSP material, B- HCl treated MOSP, C- NaCl treated MOSP, D – NaOH treated MOSP, E – water treated MOSP)

**(C))**


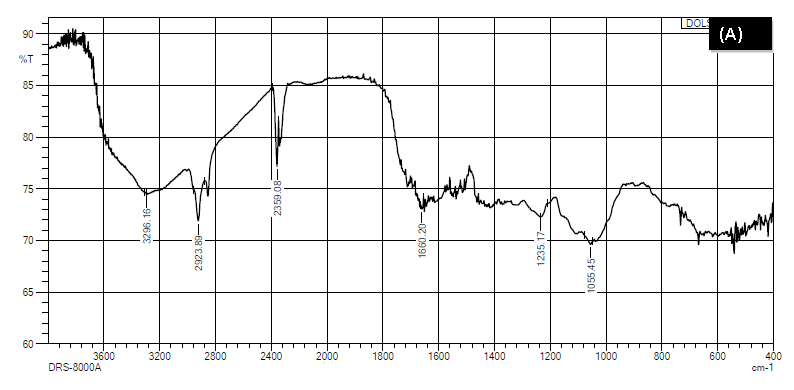


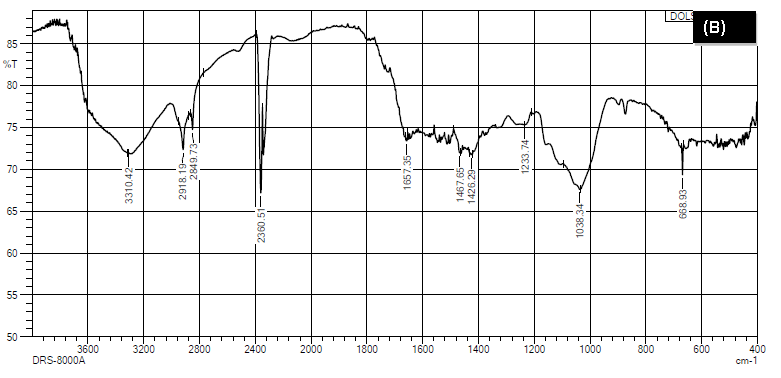


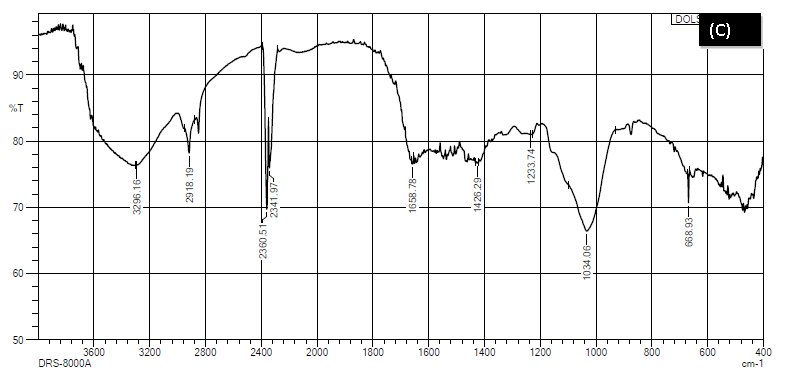


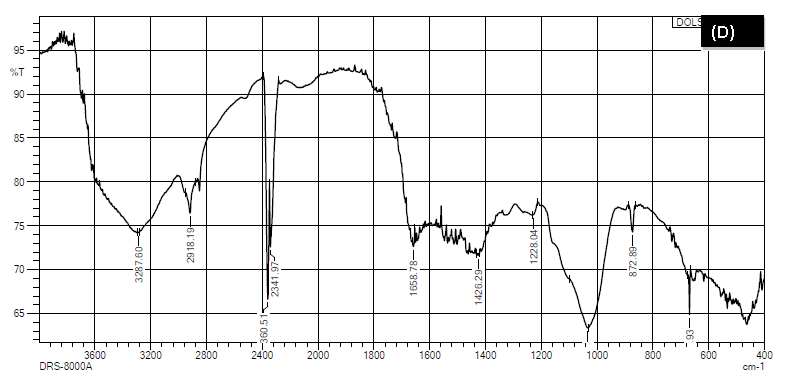


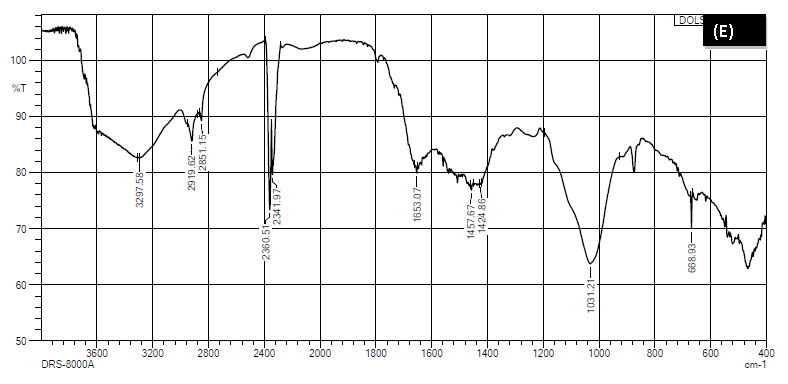


**Fig. S3**FT-IR analysis for elemental composition of MOSP sample (A- raw MOSP material, B- HCl treated MOSP, C- NaCl treated MOSP, D – NaOH treated MOSP, E – water treated MOSP)

.
